# Cyclooxygenase 2 is up-regulated and localized to macrophages in the intestine of Min mice

**DOI:** 10.1038/sj.bjc.6690224

**Published:** 1999-03

**Authors:** M A Hull, J K Booth, A Tisbury, N Scott, C Bonifer, P L Markham, P L Coletta

**Affiliations:** Molecular Medicine Unit, University of Leeds, Leeds, UK; Department of Histopathology, St James's University Hospital, Leeds, LS9 7TF, UK

**Keywords:** adenoma, cyclooxygenase, immunohistochemistry, macrophage, Min mouse, prostaglandin

## Abstract

Expression of cyclooxygenase 2 (COX-2) is believed to play an important role in adenoma formation in murine polyposis models, and inhibition of COX-2 activity may, at least, partly explain the chemopreventative activity of non-steroidal anti-inflammatory drugs against colorectal cancer in humans. However, the mechanism by which COX-2 acts in intestinal tumorigenesis remains unresolved because of conflicting data on the cellular localization of COX-2 in intestinal mucosa. Using immunohistochemistry with specific COX-2 antiserum, we have shown that COX-2 protein is localized to interstitial cells at the base of and within adenomas of the small and large intestine of multiple intestinal neoplasia (Min) mice. No COX-2 staining was observed in dysplastic epithelial cells within adenomas or in histologically normal epithelium. Moreover, COX-2 staining was observed in lamina propria cells of histologically normal intestine of Min mice. No staining was demonstrated in wild-type littermates. The rat monoclonal antibody F4/80 was used to show that COX-2-positive cells represented a subset of the macrophage population present in the intestine of Min mice. Localization of COX-2 to macrophages implies a paracrine effect of COX-2 function on epithelial cells in adenomas and also on histologically normal epithelium. Up-regulation of COX-2 expression in lamina propria macrophages may precede loss of the second functional *Apc* allele in epithelial cells before adenoma formation in the Min mouse model of intestinal tumorigenesis. © 1999 Cancer Research Campaign


*British Journal of Cancer* (1999) **79**(9/10), 1399–1405
© 1999 Cancer Research Campaign
Article no. bjoc.1998.0224

### Cyclooxygenase 2 is up-regulated and localized to macrophages in the intestine of Min mice

**MA Hull[1], JK Booth[2], A Tisbury[1], N Scott[2], C Bonifer[1], AF Markham[1] and PL Coletta[1]**

[1]Molecular Medicine Unit, University of Leeds; [2]Department of Histopathology, St James's University Hospital, Leeds LS9 7TF, UK

**Summary** Expression of cyclooxygenase 2 (COX-2) is believed to play an important role in adenoma formation in murine polyposis models, and inhibition of COX-2 activity may, at least, partly explain the chemopreventative activity of non-steroidal anti-inflammatory drugs against colorectal cancer in humans. However, the mechanism by which COX-2 acts in intestinal tumorigenesis remains unresolved because of conflicting data on the cellular localization of COX-2 in intestinal mucosa. Using immunohistochemistry with specific COX-2 antiserum, we have shown that COX-2 protein is localized to interstitial cells at the base of and within adenomas of the small and large intestine of multiple intestinal neoplasia (Min) mice. No COX-2 staining was observed in dysplastic epithelial cells within adenomas or in histologically normal epithelium. Moreover, COX-2 staining was observed in lamina propria cells of histologically normal intestine of Min mice. No staining was demonstrated in wild-type littermates. The rat monoclonal antibody F4/80 was used to show that COX-2-positive cells represented a subset of the macrophage population present in the intestine of Min mice. Localization of COX-2 to macrophages implies a paracrine effect of COX-2 function on epithelial cells in adenomas and also on histologically normal epithelium. Up-regulation of COX-2 expression in lamina propria macrophages may precede loss of the second functional *Apc* allele in epithelial cells before adenoma formation in the Min mouse model of intestinal tumorigenesis.

**Keywords:** adenoma; cyclooxygenase; immunohistochemistry; macrophage; Min mouse; prostaglandin

Data from multiple colon carcinogenesis studies in animals and human epidemiological observations suggest that non-steroidal anti-inflammatory drugs (NSAIDs) have chemopreventative activity against colorectal cancer (Weiss and Forman, 1996; Levy, 1997; Williams et al, 1997). A possible molecular target of NSAIDs is believed to be cyclooxygenase 2 (COX-2 or prostaglandin G/H synthase 2) (Levy, 1997; Williams et al, 1997). The constitutive isoform of COX, COX-1, and the mitogen-inducible isoform COX-2 both synthesize prostaglandin (PG)$H_2$ ($PGH_2$) from arachidonic acid, which, in turn, is converted to other prostaglandin (PG) species depending on the profile of PG synthases in a given cell (Levy, 1997). Exogenous $PGE_2$ stimulates proliferation of both human colon cancer cell lines and normal mouse colonocytes (Qiao et al, 1995), and $PGE_2$ levels are increased in human carcinomas compared with normal colonic mucosa (Rigas et al, 1993). Expression of COX-2 (but not COX-1) is up-regulated in human colorectal adenomas and carcinomas (Eberhart et al, 1994; Kargman et al, 1995; Sano et al, 1995; Gustafson-Svard et al, 1996; Kutchera et al, 1996) and overexpression of COX-2 in rat intestinal epithelial cells is associated with a malignant phenotype (Tsuji and DuBois, 1995). Furthermore, it has recently been reported that administration of a specific COX-2 inhibitor prevents azoxymethane-induced colon carcinogenesis in rats (Kawamori et al, 1998).

*Received 27 May 1998*
*Revised 28 July 1998*
*Accepted 29 July 1998*

*Correspondence to:* MA Hull, Division of Medicine, Clinical Sciences Building, St James's University Hospital, Leeds LS9 7TF, UK

The effects of NSAIDs on colorectal adenoma formation and growth in patients with familial adenomatous polyposis (FAP) and corresponding murine models of this condition have been particularly illuminating. Patients with FAP are heterozygous for a germline *APC* (adenomatous polyposis coli) mutation which produces truncated APC protein (Nishisho et al, 1991). Loss of the second *APC* allele leads to adenoma formation (Levy et al, 1994). Sulindac (a NSAID and non-specific COX inhibitor) reduces polyp formation in FAP patients (Williams et al, 1997). Two murine polyposis models have been studied extensively. The multiple intestinal neoplasia (Min) mutation is a heterozygous nonsense mutation at codon 850 of the *Apc* gene (Moser et al, 1990; Su et al, 1992). After somatic mutation of the second *Apc* allele, Min mice develop multiple polyps or adenomas in the small intestine as well as a small number of colonic adenomas at around 2–3 months of age (Moser et al, 1990). Adenoma formation is attenuated by sulindac (Boolbol et al, 1996; Chiu et al, 1997) and other NSAIDs such as piroxicam (Jacoby et al, 1996). The $Apc^{\Delta716}$ knockout mouse has a truncating mutation at codon 716 of the *Apc* gene and also develops adenomas in the small and large intestine (Oshima et al, 1995). The important role of COX-2 in adenoma formation in this model has been demonstrated by the highly significant reduction in polyposis in $Apc^{\Delta716}$ mice which were treated with a selective COX-2 inhibitor or had a deletion of one or both copies of the *Ptgs2* (murine COX-2) gene (Oshima et al, 1996).

Despite evidence that COX-2 overexpression is an important, early event in murine adenoma formation and that NSAIDs have a profound effect on adenoma formation, the cellular localization of COX-2 protein in adenomas remains unresolved because conflicting data have been obtained by several groups (Boolbol et al, 1996; Oshima et al, 1996; Williams et al, 1996). Because the cellular source of COX-2 in intestinal mucosa has profound implications for the mechanism by which COX-2 overexpression promotes early tumorigenesis, we investigated COX-2 localization in intestinal mucosa of Min mice by immunohistochemistry.

### MATERIALS AND METHODS

### Animals

C57BL/6J-*Apc^Min^* (Min) mice and wild-type littermates (+/+) were obtained at 6–8 weeks of age from The Jackson Laboratory (Bar Harbor, ME, USA). Male *Min*/+ animals were mated with +/+ females and the offspring genotyped using allele-specific polymerase chain reaction (PCR) (Dietrich et al, 1993). All animals were reared under isolator conditions and were shown to be pathogen free by bacteriological and serological testing. Animals were fed with mouse standard diet (B&K Universal, Hull, UK) and had free access to water. Animals were killed at specified times using carbon dioxide inhalation. Immediately after death, the gastrointestinal tract from duodenum to distal colon was obtained and cut longitudinally to expose the entire mucosal surface. Mucosa was washed thoroughly with phosphate-buffered saline (PBS) and small intestinal and colonic tumours were counted systematically by naked-eye examination. The smallest countable tumours were 1 mm in diameter.

### Immunohistochemistry

Specimens of intestine approximately 5–10 mm in length were obtained from small intestine and colon of Min mice and wild-type littermates immediately after death. Specimens were either fixed in 4% paraformaldehyde overnight before embedding in paraffin or were embedded in OCT compound (Merck, Poole, UK) and frozen at – 70°C.

Paraformaldehyde-fixed sections (3 μm thick) were mounted on glass slides coated with 3-aminopropyltriethoxysilane. Sections were dewaxed in xylene (2 × 5 min) and rehydrated with 100% ethanol followed by distilled water. Endogenous peroxidase activity was blocked using 0.6% hydrogen peroxide in methanol for 10 min. After washing with water, sections were placed in 10 mmol $l^{-1}$ citrate buffer, pH 6.0, and heated to 80°C for 10 min in a microwave oven. Non-specific binding sites were blocked with 5% swine serum (Dako, High Wycombe, UK) for 10 min at room temperature.

Mouse COX-2 antiserum (Cayman Chemical, Ann Arbor, MI, USA) was generated in rabbits by immunization with a synthetic 17-mer peptide (CY-SHSRLDDINPTVLIK) corresponding to a unique C-terminal sequence in murine COX-2 (residues 584–598) which is not present in COX-1 (Regier et al, 1993). This antiserum was specific for COX-2 (rather than COX-1) when tested by Western blot analysis (Otto and Smith, 1994).

Antiserum was diluted 1:100 in 5% swine serum and incubated with sections for 60 min at room temperature. After washes with water, sections were incubated with a 1:200 dilution of biotinylated swine anti-rabbit immunoglobulin antibody (Dako) for 30 min at room temperature, washed in tris-HCl, pH 7.8, and visualized using streptavidin–biotin–horseradish peroxidase (Dako) and 3,3′-diaminobenzidene (0.7 mg $ml^{-1}$)/hydrogen peroxide (0.05%). Finally, sections were counterstained with Mayer's haematoxylin, dehydrated and mounted in diphenylxylene (Merck). Adjacent sections were stained with haematoxylin and eosin (H & E).

Controls involved omission of the primary antiserum, incubation with an equivalent concentration of preimmune rabbit serum (Dako) and preabsorption of antiserum with 10 μg $ml^{-1}$ of its cognate 17-mer peptide (Cayman Chemical) for 2 h at 4°C before incubation with sections.

Frozen sections (8 μm thick) were fixed in 100% acetone for 60 s and air dried before immunohistochemistry, which was carried out as described above.

Adjacent paraformaldehyde-fixed sections of Min mouse small intestine were also stained with the rat monoclonal $IgG_{2b}$ antibody F4/80 (a gift from Dr P Leenen, Erasmus University, Rotterdam, The Netherlands) which recognizes mature murine macrophages (Austyn and Gordon, 1981). The method used was as described above except that there was no pretreatment of sections before blocking non-specific binding sites and incubation of sections with F4/80 was carried out overnight at 4°C. Biotinylated rabbit anti-rat immunoglobulin antibody preabsorbed with mouse immunoglobulin was obtained from Dako.

### RESULTS

Min mice were killed on day 157 ± 23 (mean ± s.e.m.; $n$ = 6) and wild-type mice on day 221 ± 12 ($n$ = 4). The number of adenomas in *Min*/+ animals was similar to that previously described (Moser et al, 1990; Boolbol et al, 1996). No adenomas were detected in wild-type littermates.

Small intestinal and colonic adenomas from Min mice were easily identifiable on H & E sections as raised lesions, 0.5–2.0 mm in length, containing dysplastic epithelial cells above a base of connective tissue and interstitial cells. The luminal surface of adenomas was often eroded with some epithelial cell necrosis. Neighbouring Min intestinal mucosa had normal morphology with intact crypt and villus architecture and with no apparent increase in lamina propria cell density compared with wild-type mucosa.

Immunohistochemistry for COX-2 revealed localization of COX-2 protein in clusters of lamina propria cells at the base of small intestinal adenomas (Figure 1A and B). Staining for COX-2 was also demonstrated in interstitial cells within adenomas (Figure 1D), but there was no localization of COX-2 in dysplastic epithelial cells within adenomas (Figure 1A and D). Interstitial cells also stained for COX-2 in close proximity to areas of eroded adenoma surface (Figure 1E). Although superficial epithelial cells stained for COX-2 (Figure 1E), this was shown to be non-specific (Figure 1F).

Examination of adjacent sections stained with H & E suggested that the majority of COX-2-positive cells were macrophages (speckled nuclear chromatin staining and irregular cell shape). This was confirmed by immunohistochemistry with the mouse macrophage marker F4/80 (Figure 1G and H).

A proportion of F4/80-positive cells in the lamina propria of histologically normal small intestinal mucosa, at least 5 mm distant from any adenoma, also stained for COX-2 (Figures 1J, L and M), although, subjectively, the number of COX-2-positive cells seemed lower than at the base of adenomas and many F4/80-positive cells did not contain immunoreactive COX-2. By contrast, lamina propria cells from small intestine of wild-type mice of a similar age did not stain for immunoreactive COX-2 (Figure 1Q).

Less staining for COX-2 was evident in colonic mucosa of Min mice than small intestine. In colonic adenomas, COX-2 staining

 © Cancer Research Campaign 1999

A B

C D

E F

G H

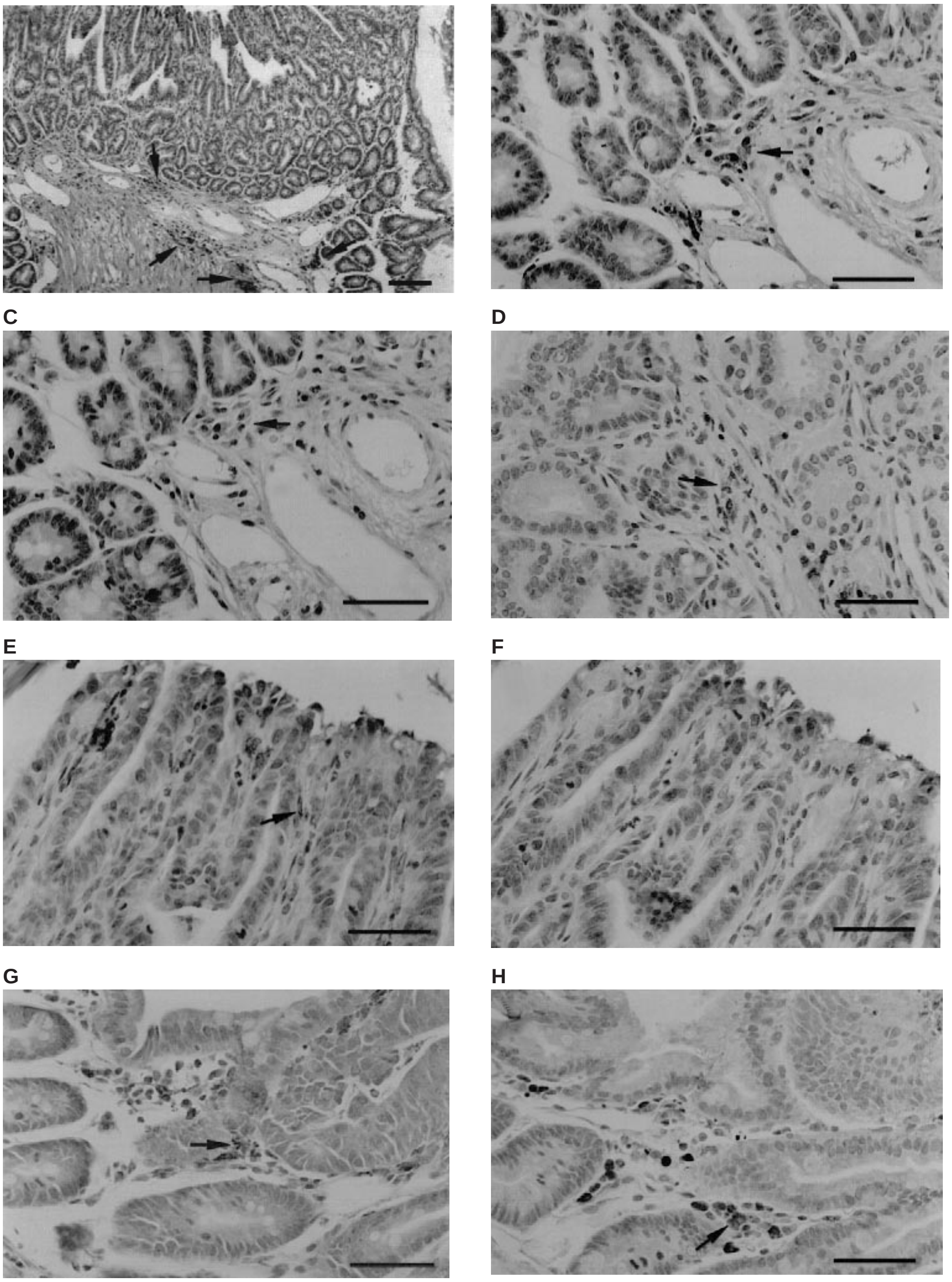

**Figure 1** COX-2 localization in Min (*Min*/+) and wild-type (+/+) mouse intestine. (**A**) *Min*/+ small intestinal adenoma. COX-2 was localized predominantly to lamina propria cells at the base of the adenoma (arrows). Dysplastic epithelial cells did not stain for COX-2. Bar = 80 μm. (**B**) *Min*/+ small intestinal adenoma. Localization of COX-2 in cells at the base of an adenoma (arrow). Bar = 40 μm. (**C**) *Min*/+ small intestinal adenoma. Staining of cells at the base of an adenoma (arrow; compare with **B**) was abolished after preabsorption of COX-2 antiserum with its cognate peptide. Bar = 40 μm. (**D**) *Min*/+ small intestinal adenoma. Localization of COX-2 in interstitial cells within an adenoma (arrow). Epithelial cells did not stain for COX-2. Bar = 40 μm. (**E**) *Min*/+ small intestinal adenoma. Eroded epithelium at the luminal surface of an adenoma. COX-2 was localized to interstitial cells just below the erosion and within the body of the adenoma (arrow). Bar = 40 μm. (**F**) *Min*/+ small intestinal adenoma. Abolition of staining of interstitial cells after preabsorption of COX-2 antiserum with COX-2 peptide (compare with **E**). Staining of superficial epithelial cells was non-specific. Bar = 40 μm. (**G**) and (**H**) *Min*/+ small intestinal adenoma. F4/80-positive cells at the base of an adenoma (arrow; **G**). These cells co-stained for COX-2 (arrow; **H**). Bar = 40 μm. (**J**) *Min*/+ normal small intestine. COX-2 was localized to lamina propria cells within and at the base of villi (arrows). Bar = 80 μm. (**K**) *Min*/+ normal small intestine. Abolition of staining after preabsorption with COX-2 peptide (arrows; see **J**). Bar = 80 μm. (**L**) *Min*/+ normal small intestine. COX-2 was localized to lamina propria cells within villi (arrows). Bar = 40 μm. (**M**) *Min*/+ normal small intestine. F4/80 staining confirmed that COX-2-positive cells were macrophages. Bar = 40 μm. (**N**) *Min*/+ normal small intestine. Staining of frozen sections confirmed the localization of COX-2 in lamina propria cells (arrows). Bar = 40 μm. (**P**) *Min*/+ normal small intestine. Abolition of specific COX-2 staining of frozen sections after preabsorption with COX-2 peptide (arrows; see **N**). Bar = 40 μm. (**Q**) +/+ small intestine. No COX-2 staining of lamina propria cells was seen (compare with **J**). Bar = 80 μm. (**R**) *Min*/+ colonic adenoma. Localization of COX-2 to interstitial cells within a colonic adenoma (arrows). Epithelial cells did not stain for COX-2. Bar = 80 μm. (**S**) *Min*/+ normal colon. Very few lamina propria cells stained for COX-2 (arrow). The diffuse staining of superficial epithelial cells was non-specific. Bar = 40 μm

© Cancer Research Campaign 1999 

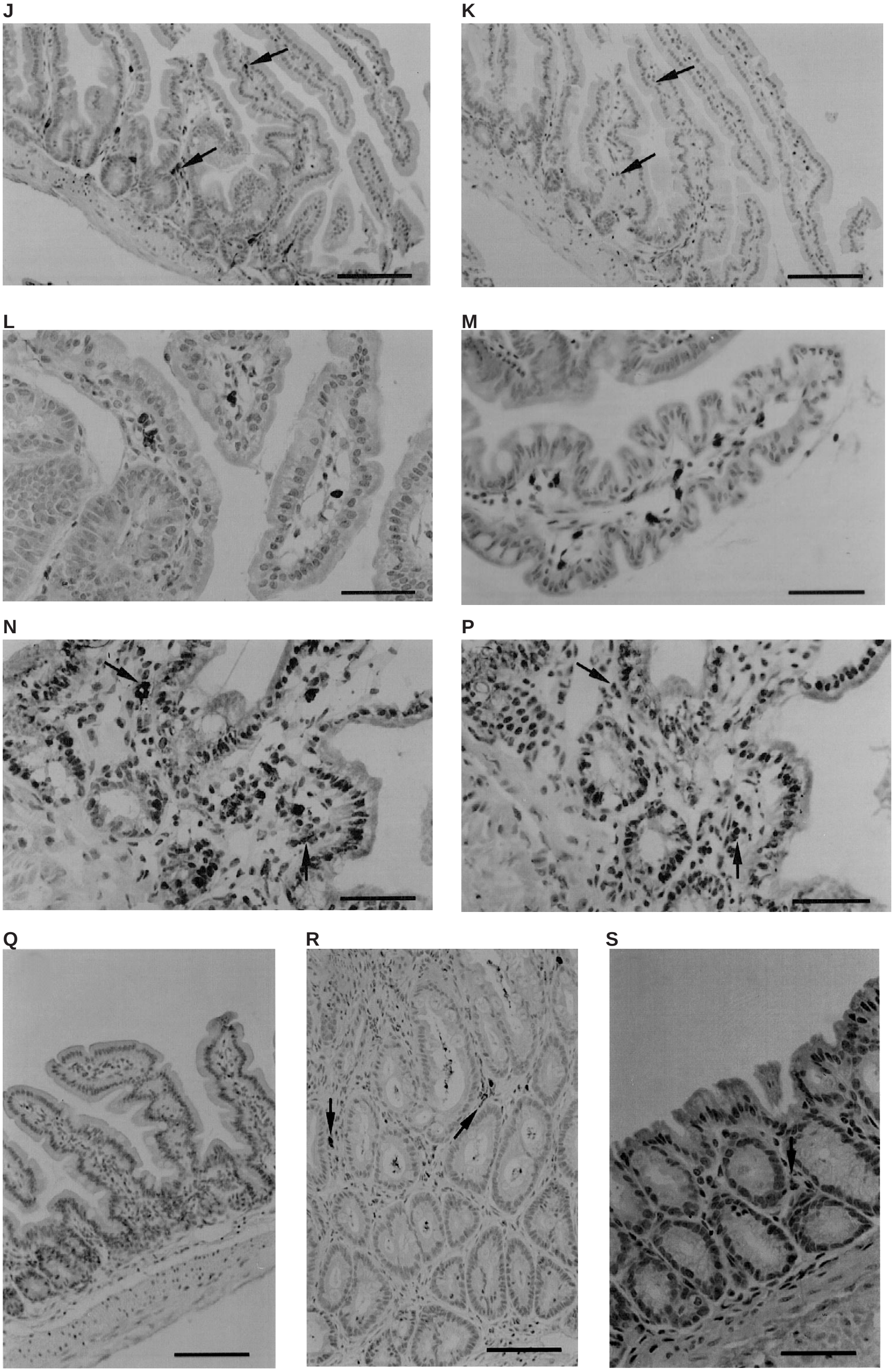

**Figure 1 (cont)**

was detected in a similar population of interstitial cells (Figure 1R), but COX-2-positive cells were fewer in number than in small intestinal adenomas. Dysplastic epithelial cells of colonic adenomas were clearly negative for COX-2 (Figure 1R). In contrast to small intestine, histologically normal Min mouse colonic mucosa contained only few COX-2-positive lamina propria cells (Figure 1S). No COX-2 immunoreactivity was detected in colonic mucosa from wild-type mice.

© Cancer Research Campaign 1999

The control sections confirmed the specificity of the observed staining for COX-2. Preabsorption of COX-2 antiserum with the COX-2 peptide abolished staining of interstitial cells in adenomas (Figure 1C and F) and lamina propria cells in adjacent histologically normal small intestinal mucosa (Figure 1K). Additionally, control sections incubated without primary antiserum or with preimmune rabbit serum did not show any staining. We also examined frozen sections of Min mouse intestine to confirm our findings on fixed tissue sections. Immunohistochemistry for COX-2 confirmed that there was no staining of adenomatous epithelium, but there was staining of lamina propria cells (Figure 1N) which was again prevented by preabsorption with COX-2 peptide (Figure 1P).

### DISCUSSION

We have demonstrated that COX-2 protein is localized to interstitial cells (but not epithelial cells) in intestinal adenomas and also to lamina propria cells in histologically normal intestinal mucosa of Min mice of a similar age and phenotype to those previously described (Moser et al, 1990, 1992; Dietrich et al, 1993; Boolbol et al, 1996). In contrast, COX-2 immunostaining was not seen in mucosa from wild-type littermates. Morphological analysis of H & E sections suggested that the majority of COX-2-positive cells were macrophages and this was confirmed by staining with F4/80 which recognizes all mature tissue macrophages in the mouse (Austyn and Gordon, 1981). This finding is consistent with the study of Kargman et al (1996) which demonstrated restriction of COX-2 localization to a subset of macrophages in rat intestine. The distribution of macrophages, which stained for F4/80, in small intestine in this study is similar to that previously reported (Hume et al, 1984; Soesatyo et al, 1990). Not all F4/80-positive cells co-localized COX-2 protein. As activation of cultured mouse macrophages is associated with up-regulation of COX-2 expression (Lee et al, 1992), it is possible that COX-2 expression in small intestine of Min mice is restricted to activated macrophages. Further careful immunotyping and double labelling studies are required to answer this question.

Our data on COX-2 localization in small intestinal adenomas are in agreement with complimentary studies by Oshima et al (1996), who reported that COX-2 was expressed in interstitial cells within adenomas of $Apc^{\Delta 716}$ mice using immunohistochemistry for β-galactosidase to localize *lacZ* expression under the control of the *ptgs2* promoter. However, conflicting data have been provided by Williams et al (1996), who described immunostaining for COX-2 in the adenomatous epithelium of a small number of polyps from B6 × 129 *Min*/+ mice. These authors confirmed the specificity for COX-2 (rather than COX-1) of their antibody, using cultured intestinal epithelial cell lines, but did not provide controls confirming the specificity of staining for COX-2 in fixed tissue sections which had been predigested with trypsin.

Restriction of COX-2 localization to interstitial macrophages implies the possibility of a direct and/or indirect paracrine effect of COX-2-derived eicosanoid products on neighbouring epithelial cells in adenomas. The nature of any putative macrophage–epithelial cell signal is unknown, but eicosanoids such as $PGE_2$ promote proliferation of cultured human colorectal cancer cells (Qiao et al, 1995). It is also recognized that certain murine tumour-derived macrophage cell lines synthesize large amounts of $PGD_2$ (McGuire et al, 1985), whose dehydration products, the $PGJ_2$ series, could alter gene expression by binding to the peroxisome proliferator-activated receptor (PPAR) family of nuclear transcription factors (Kliewer et al, 1995). Recently, PPARγ expression has been shown to be up-regulated in azoxymethane-induced colonic adenomas and carcinomas in rats as well as a subset of human colon cancer cell lines (DuBois et al, 1998).

We have also shown that lamina propria macrophages in small intestinal mucosa of Min mice distant from any adenoma also contain immunoreactive COX-2, unlike corresponding mucosa from wild-type littermates. This finding is in agreement with data provided by Boolbol et al (1996), who demonstrated increased COX-2 expression (by Western blot analysis), increased eicosanoid levels and decreased epithelial cell apoptosis in histologically normal small intestine of Min mice at 110 days compared with wild-type intestine. This study did not include immunohistochemical detection of COX-2. However, Western blot analysis of normal small intestinal mucosa of $Apc^{\Delta 716}$ mice failed to detect COX-2 (Oshima et al, 1996), and the one previous immunohistochemical study of COX-2 in normal small intestine of B6 × 129 *Min*/+ mice failed to detect COX-2 (Williams et al, 1996). Additional backcross experiments are required to investigate whether intestinal COX-2 localization is related to the genetic background of *Min*/+ mice. It is possible that the presence or absence of COX-2 expression in certain mouse strains could modify tumour phenotype in *Min*/+ mice, as has previously been described for secretory type II phospholipase A2 (the gene product of the *Mom-1* locus) (Dietrich et al, 1993; MacPhee et al, 1995).

Although it has been suggested that induction of COX-2 expression in murine *Apc* +/– polyposis models occurs downstream of the loss of the second *Apc* allele in epithelial cells (Prescott and White, 1996), our data demonstrate the presence of COX-2 in macrophages in close proximity to epithelial cells which appear morphologically normal and which, presumably, possess a full-length *Apc* allele. Two previous studies have demonstrated that histologically normal intestinal epithelium from Min mice contains cells which have not undergone somatic mutation of their second *Apc* gene (Levy et al, 1994; Luongo et al, 1994). Therefore, it is unlikely that we have examined normal epithelium in the period between loss of *Apc* heterozygosity and neoplastic transformation.

A recent study has demonstrated that histologically normal Min mouse epithelium has decreased proliferation, apoptosis and crypt–villus migration rates compared with wild-type mouse epithelium (Mahmoud et al, 1997), which suggests a dominant-negative effect of heterozygous *Apc* mutation on epithelial cells. Up-regulation of COX-2 expression in neighbouring lamina propria cells could also be a primary dominant-negative effect of heterozygous *Apc* mutation in these cells or alternatively it could be secondary to a stimulatory signal from *Apc* +/– epithelial cells. There are also alterations in proliferative activity of histologically normal colonic epithelium in FAP patients (Mills et al, 1995), and so it would be interesting to assess whether COX-2 is localized to lamina propria macrophages adjacent to histologically normal epithelium in these patients.

An alternative explanation for the localization of COX-2 in lamina propria cells next to histologically normal intestinal epithelium is a ‘field effect’ of adenomas in Min mouse intestine. Mahmoud et al (1997) ruled this out as a cause of changes in epithelial cell kinetics by examining Min mouse intestine at 5 weeks of age (before adenoma formation). Our preliminary data are consistent with this finding and confirm that COX-2 is expressed by lamina propria macrophages in histologically normal intestine of Min mice before adenoma formation (data not shown).

© Cancer Research Campaign 1999

In keeping with these findings, it has recently been reported (Singh et al, 1997) that colonic COX-2 expression is up-regulated as early as 1 week after azoxymethane administration in rats (which is several weeks before tumour formation in this model).

Localization of COX-2 in Min mouse colon has not been reported previously. The distribution of COX-2 immunostaining was similar to that in small intestinal adenomas and histologically normal mucosa, although COX-2-positive cells were much less frequent in colonic mucosa. This mirrors the distribution of macrophages in normal mouse intestine (Hume et al, 1984) and could explain the relative paucity of adenomas in the colon of Min mice compared with small intestine (which contrasts with the FAP phenotype).

There has been no published study of the immunohistochemical localization of COX-2 in human adenomas. However, Eberhart et al (1994) have reported that 9 out of 20 sporadic adenomas had increased COX-2 mRNA expression by Northern blot analysis. Another study failed to detect COX-2 protein by Western blot analysis in any of four small FAP adenomas (Kargman et al, 1995). Therefore, it is possible that there is a similar localization of COX-2 in human sporadic and FAP adenomas to that demonstrated in this study.

In summary, we have shown that COX-2 is localized to lamina propria macrophages in intestinal adenomas and histologically normal intestinal mucosa of Min mice but not wild-type littermates. The mechanism by which COX-2 expression is involved in adenoma formation and growth is not known, but may involve paracrine signalling by COX-2-derived eicosanoids from macrophages to neighbouring epithelial cells. The elucidation of the role of COX-2 in adenoma formation may lead to development of alternative strategies for chemoprevention of colorectal cancer which are not complicated by the morbidity and mortality which is associated with NSAID use.

### ACKNOWLEDGEMENTS

This work was funded by grants from Yorkshire Cancer Research and The Medical Research Council.

This work has been previously presented at the British Society of Gastroenterology Annual Meeting in Harrogate, 10–13 March 1998 [*Gut* **42** (suppl. 1). A53] and the American Gastroenterological Association meeting in New Orleans, LA, 17–20 May 1998.

### REFERENCES

Austyn JM and Gordon S (1981) F4/80, a monoclonal antibody directed specifically against the mouse macrophage. *Eur J Immunol* **11**: 805–815

Boolbol SK, Dannenberg AJ, Chadburn A, Martucci C, Guo X, Ramonetti JT, Abreu-Goris M, Newmark HL, Lipkin ML, DeCosse JJ and Bertagnolli MM (1996) Cyclooxygenase-2 overexpression and tumor formation are blocked by sulindac in a murine model of familial adenomatous polyposis. *Cancer Res* **56**: 2556–2560

Chiu C-H, McEntee MF and Whelan J (1997) Sulindac causes rapid regression of preexisting tumors in *Min*/+ mice independent of prostaglandin biosynthesis. *Cancer Res* **57**: 4267–4273

Dietrich WF, Lander ES, Smith JS, Moser AR, Gould KA, Luongo C, Borenstein N and Dove W (1993) Genetic identification of *Mom-1*, a major modifier locus affecting *Min*-induced intestinal neoplasia in the mouse. *Cell* **75**: 631–639

DuBois RN, Gupta R, Brockman J, Reddy BS, Krakow SL and Lazar MA (1998) The nuclear eicosanoid receptor, PPARγ, is aberrantly expressed in colonic cancers. *Carcinogenesis* **19**: 49–53

Eberhart CE, Coffey RJ, Radhika A, Giardiello FM, Ferrenbach S and DuBois RN (1994) Up-regulation of cyclooxygenase 2 gene expression in human colorectal adenomas and adenocarcinomas. *Gastroenterology* **107**: 1183–1188

Gustafson-Svard C, Lilja I, Hallbook O and Sjodahl R (1996) Cyclooxygenase-1 and cyclooxygenase-2 gene expression in human colorectal adenocarcinomas and in azoxymethane induced colonic tumours in rats. *Gut* **38**: 79–84

Hume DA, Perry VH and Gordon S (1984) The mononuclear phagocyte system of the mouse defined by immunohistochemical localisation of antigen F4/80: macrophages associated with epithelia. *Anat Record* **210**: 503–512

Jacoby JF, Marshall DJ, Newton MA, Novakovic K, Tutsch K, Cole CE, Lubet RA, Kelloff GJ, Verma A, Moser AR and Dove WF (1996) Chemoprevention of spontaneous intestinal adenomas in the *Apc*$^{Min}$ mouse model by the nonsteroidal anti-inflammatory drug piroxicam. *Cancer Res* **56**: 710–714

Kargman SL, O'Neill GP, Vickers PJ, Evans JF, Mancini JA and Jothy S (1995) Expression of prostaglandin G/H synthase-1 and -2 protein in human colon cancer. *Cancer Res* **55**: 2556–2559

Kargman S, Charleson S, Cartwright M, Frank J, Riendeau D, Mancini J, Evans J and O'Neill G (1996) Characterization of prostaglandin G/H synthase 1 and 2 in rat, dog, monkey, and human gastrointestinal tracts. *Gastroenterology* **111**: 445–454

Kawamori T, Rao CV, Seibert K and Reddy BS (1998) Chemopreventative activity of celecoxib, a specific cyclooxygenase-2 inhibitor, against colon carcinogenesis. *Cancer Res* **58**: 409–412

Kliewer SA, Lenhard JM, Willson TM, Patel I, Morris DC and Lehmann JM (1995) A prostaglandin $J_2$ metabolite binds peroxisome proliferator-activated receptor γ and promotes adipocyte differentiation. *Cell* **83**: 813–819

Kutchera W, Jones DA, Matsunami N, Groden J, McIntyre TM, Zimmerman GA, White RL and Prescott SM (1996) Prostaglandin H synthase 2 is expressed abnormally in human colon cancer: evidence for a transcriptional effect. *Proc Natl Acad Sci USA* **93**: 4816–4820

Lee SH, Soyoola E, Chanmugam P, Hart S, Sun W, Zhong H, Liou S, Simmons D and Hwang D (1992) Selective expression of mitogen-inducible cyclooxygenase in macrophages stimulated with lipopolysaccharide. *J Biol Chem* **267**: 25934–25938

Levy GN (1997) Prostaglandin H synthases, nonsteroidal anti-inflammatory drugs, and colon cancer. *FASEB J* **11**: 234–247

Levy DB, Smith KJ, Beazer-Barclay Y, Hamilton SR, Vogelstein B and Kinzler KW (1994) Inactivation of both *APC* alleles in human and mouse tumors. *Cancer Res* **54**: 5953–5958

Luongo C, Moser AR, Gledhill S and Dove WF (1994) Loss of *Apc*$^{+}$ in intestinal adenomas from Min mice. *Cancer Res* **54**: 5947–5952

MacPhee M, Chepenik KP, Liddell RA, Nelson KK, Siracusa LD and Buchberg AM (1995) The secretory phospholipase A2 gene is a candidate for the *Mom1* locus, a major modifier of *Apc*$^{Min}$-induced intestinal neoplasia. *Cell* **81**: 957–966

Mahmoud NN, Boolbol SK, Bilinski RT, Martucci C, Chadburn A and Bertagnolli MM (1997) *Apc* gene mutation is associated with a dominant-negative effect upon intestinal cell migration. *Cancer Res* **57**: 5045–5050

McGuire JC, Richard KA, Sun FF and Tracey DE (1985) Production of prostaglandin $D_2$ by murine macrophage cell lines. *Prostaglandins* **30**: 949–967

Mills SJ, Shepherd NA, Hall PA, Hastings A, Mathers JC and Gunn A (1995) Proliferative compartment deregulation in the non-neoplastic colonic epithelium of familial adenomatous polyposis. *Gut* **36**: 391–394

Moser AR, Pitot HC and Dove WF (1990) A dominant mutation that predisposes to multiple intestinal neoplasia in the mouse. *Science* **247**: 322–324

Moser AR, Dove WF, Roth KA and Gordon JI (1992) The *Min* (multiple intestinal neoplasia) mutation: its effect on gut epithelial cell differentiation and interaction with a modifier system. *J Cell Biol* **116**: 1517–1526

Nishisho I, Nakamura Y, Miyoshi Y, Miki Y, Ando H, Horii A, Koyama K, Utsunomiya J, Baba S, Hedge P, Markham A, Krush AJ, Petersen G, Hamilton SR, Nilbert MC, Levy DB, Bryan TM, Preisinger AC, Smith KJ, Su L-K, Kinzler KW and Vogelstein B (1991) Mutations of chromosome 5q21 genes in FAP and colorectal cancer patients. *Science* **253**: 665–669

Oshima M, Oshima H, Kitagawa K, Kobayashi M, Itakura C and Taketo M (1995) Loss of *Apc* heterozygosity and abnormal tissue building in nascent intestinal polyps in mice carrying a truncated *Apc* gene. *Proc Natl Acad Sci USA* **92**: 4482–4486

Oshima M, Dinchuk JE, Kargman SL, Oshima H, Hancock B, Kwong E, Trzaskos JM, Evans JF and Taketo MM (1996) Suppression of intestinal polyposis in *Apc*$^{\Delta 716}$ knockout mice by inhibition of cyclooxygenase 2 (COX-2). *Cell* **87**: 803–809

Otto JC and Smith WL (1994) The orientation of prostaglandin endoperoxide synthases-1 and -2 in the endoplasmic reticulum. *J Biol Chem* **269**: 19868–19875

Prescott SM and White RL (1996) Self-promotion? Intimate connections between APC and prostaglandin H synthase-2. *Cell* **87**: 783–786

Qiao L, Kozoni V, Tsioulias GJ, Koutsos MI, Hanif R, Shiff SJ and Rigas B (1995) Selected eicosanoids increase the proliferation rate of human colon

 *© Cancer Research Campaign 1999*

carcinoma cell lines and mouse colonocytes in vivo. *Biochim Biophys Acta* **258**: 215–223

Regier MK, DeWitt DL, Schindler MS and Smith WL (1993) Subcellular localization of prostaglandin endoperoxide synthase-2 in murine 3T3 cells. *Arch Biochem Biophys* **301**: 439–444

Rigas B, Goldman IS and Levine L (1993) Altered eicosanoid levels in human colon cancer. *J Lab Clin Med* **122**: 518–523

Sano H, Kawahito Y, Wilder RL, Hashiramoto A, Mukai S, Asai K, Kimura S, Kato H, Kondo M and Hla T (1995) Expression of cyclooxygenase-1 and -2 in human colorectal cancer. *Cancer Res* **55**: 3785–3789

Singh J, Hamid R and Reddy BS (1997) Dietary fat and colon cancer: modulation of cyclooxygenase-2 by types and amount of dietary fat during the postinitiation stage of colon carcinogenesis. *Cancer Res* **57**: 3465–3470

Soesatyo M, Biewenga J, Kraal G and Sminia T (1990) The localization of macrophage subsets and dendritic cells in the gastrointestinal tract of the mouse with special reference to the presence of high endothelial venules. *Cell Tissue Res* **259**: 587–593

Su L-K, Kinzler KW, Vogelstein B, Preisinger AC, Moser AR, Luongo C, Gould KA and Dove WF (1992) Multiple intestinal neoplasia caused by a mutation in the murine homolog of the APC gene. *Science* **256**: 668–670

Tsuji M and DuBois RN (1995) Alterations in cellular adhesion and apoptosis in epithelial cells overexpressing prostaglandin endoperoxide synthase 2. *Cell* **83**: 493–501

Weiss HA and Forman D (1996) Aspirin, non-steroidal anti-inflammatory drugs and protection from colorectal cancer: a review of the epidemiological evidence. *Scand J Gastroenterol* **31** (suppl. 220): 137–141

Williams CS, Luongo C, Radhika A, Zhang T, Lamps LW, Nanney LB, Beauchamp RD and DuBois RN (1996) Elevated cyclooxygenase-2 levels in *Min* mouse adenomas. *Gastroenterology* **111**: 1134–1140

Williams CS, Smalley W and DuBois RN (1997) Aspirin use and potential mechanisms for colorectal cancer prevention. *J Clin Invest* **100**: 1325–1329

© Cancer Research Campaign 1999
